# Evaluating the pedestrian level of service for varying trip purposes using machine learning algorithms

**DOI:** 10.1038/s41598-024-53403-7

**Published:** 2024-02-02

**Authors:** Deborah Paul, Sara Moridpour, Srikanth Venkatesan, Nuwan Withanagamage

**Affiliations:** 1 Department of Civil and Infrastructure Engineering, RMIT University, Melbourne, Australia; 2 Faculty of Information Technology, Monash University, Melbourne, Australia

**Keywords:** Civil engineering, Psychology and behaviour

## Abstract

The pedestrians’ feeling of comfort while walking on footpaths varies according to the time of day, environment, and the purpose of the trip. The quality of service offered by pedestrian facilities such as walkways, intersections, and public places is evaluated by the Pedestrian level of service (PLOS) and has been measured from time to time, to upgrade and maintain the sustainable travel choice of people. This paper aims to focus on the level of service based on three main trip purposes such as work, education, and recreation, while considering various path characteristics and pedestrian flow characteristics that affect the pedestrian’s feeling of comfort on the walkways. The data has been collected using pedestrian questionnaire surveys and pedestrian sensors in the Melbourne central business district and the significant factors that influence the PLOS for each trip purpose will be chosen using the Mutual Information gain, which is found to be different for each trip purpose. The major influencing factors that affect the PLOS will be used to develop machine learning models for three trip purposes separately using Random Forest and Light-GBM algorithm in Python. The accuracy of prediction using the light GBM model is 0.74 for education, 0.80 for recreation, and 0.70 for work trip purposes. It is found using SHAP which stands for Shapely Additive explanations that the factors such as interpersonal distance, distance from vehicles, construction sites, vehicle volume, traffic noise, and footpath surface are the most influencing variables that affect the PLOS based on three different trip purposes.

## Introduction

Walking is a sustainable mode of transport, and it is an activity performed by almost all individuals for varying distances of travel in everyday life. It is also a healthier choice of transport and has been considered as a form of active transportation in transport Infrastructure. Hence pedestrians play an important role in the infrastructure of the city. Developed countries give more importance to pedestrian facilities and invest more money to improve the future of pedestrian movement. In Melbourne, most people who travel to the city prefer to use public transport because of the limited car parking space in the city and usually walk to their respective destinations from the public transport stations. As a result, we can find an increase in pedestrian population in the city compared to suburbs away from the city, where people walk for mainly recreation purposes in residential zones. People walk for various reasons and various studies in the literature show that the walking behaviour of pedestrians is related to three different choices. Firstly, activity choice is when people want to engage themselves in some physical activity, secondly, destination choice is when people want to access amenities located at short distances by walking and finally, mode choice is when people prefer to walk outside and choose walking as the mode of travel^1^. According to the data collected by the National Health Survey during 2014–15, 50% of the adults aged 18–64, who live in major cities in Australia, have insufficient physical activity. This rate varied with the socioeconomic condition of the area and 40% of the adults living in the highest socioeconomic areas undertake insufficient physical activity including active transport^2^. The national health survey also reveals the fact that out of 40% of the adults who participate in physical activities, 16% walked for transport because walking was their only form of physical activity and only 13% walked for transport and exercise purposes^3^. Hence comfortable walking conditions will help to increase the physical activity levels of the adult groups by encouraging active transport.

Walking is a basic mode of transport that does not depend on external resources to operate and is mostly adopted for traveling short distances. A pedestrian pedestrian-friendly environment that promotes physical activity is important for a healthy infrastructure in developed countries like Melbourne. This has drawn attention to creating new measures to assess the comfort of pedestrians who use those facilities^4^. Although there has been importance placed on the trip purpose of path users in many of the research conducted in the past^5–8^, we find that it has not been used in the model to evaluate its impact on the comfort of users. The variables considered for finding pedestrian comfort also vary based on the methodology used for research and the location where the research was conducted. To address these issues, the current study has considered all possible variables that impact pedestrian comfort while walking on a footpath in central cities and also has adopted a machine learning approach to develop a model to evaluate the pedestrian level of service which could be used by city planners and local government for changes to provide an excellent walking condition in central cities and to promote walking as the mode of transport in areas where vehicle movement has to be minimum.

The paper has the following sections. The first section contains the background and relevant research works on pedestrian level of service. The section following the literature review presents the data collected through questionnaire surveys and sensors. The section after that explains the method used in this research and the machine learning algorithms used for the model. Following that, under the results and discussions, the results of the model will be explained. Finally, the future direction presents the possible expansion of the research.

## Literature review

Walkability and Pedestrian level of Service are two commonly used terms that are used to refer to the quality of service that a pedestrian can enjoy while walking. Walkability is defined as the ability of a place to connect people to various other places in less time and effort and to provide interest in their trips by offering a good visual environment^9^. The pedestrian level of service has been widely used to refer to the operational quality of pedestrian facilities such as sidewalks, footpaths, and crosswalks and it has been derived from the Level of Service concept in traffic studies which was first used in vehicles and then later developed for pedestrian facilities^10^. PLOS defined as the pedestrian flow rate and walkway capacity is taken as one of the components of Walkability^11^. Safety and security are also some important elements of walkability; empirical studies have been conducted in Australia to find the mindset of pedestrians about the risk they will undertake if they are faced with accidents causing minor or fatal injuries^12^. Further, Walkability also considers features such as continuity, safety, pleasantness, and pleasurability while walking on footpaths in an area^13,14^. The Pedestrian Level of Service measures the service quality of the pedestrian facilities by using variables more related to walkway features and pedestrian flow compared to those involved in assessing Walkability. The current research highlights the variables and methodologies that were adopted to evaluate the PLOS based on different strategies such as Capacity-based, safety-based, Comfort-based, and Quality-based models.

### Capacity-based models

In the early methods used for finding the LOS such as Fruin, the density and speed of pedestrians were recorded through time-lapse photography to find the PLOS from the different flow rates of pedestrians. The Fruin’s scale gives the standards based on the speed, ability to overtake slow-moving pedestrians, and bidirectional flow movements^15^. In the highway capacity manual (HCM), pedestrian’s speed and pedestrian unit flow rate were used to classify the different comfort levels experienced by the pedestrians which is based on a similar concept to road LOS^16^. The behaviour of pedestrians was found to be quite different compared to the behaviour of vehicles while assessing the level of service in Korea and hence factors such as personal space and evasive movements have been considered apart from pedestrian density and flow which was used by traditional methods such as HCM2000^16,17^. Multiple regression techniques were used to find the LOS for the walkway condition in Korea by collecting data through video recordings about the evasive movements of pedestrians, walkway width, personal space, flow rate, and speed of pedestrians. The thresholds for average space, flow rate, average speed, and v/c ratio in the Indian context were established using the fuzzy-GA clustering method by Sahani and Bhuyan^18^. Video recording was set up in certain locations to gather data on the pedestrian speed and flow rate. Then three major clustering techniques Affinity Propagation, Self-Organizing Map in Artificial Neural Networks, and Genetic Algorithm-Fuzzy clustering were applied to find the suitable method that had lower Wilk’s Lambda value and to establish the value ranges for average pedestrian space, speed, and flowrate in that location. Raghuwanshi and Tare^19^ developed a multiple linear regression model based on the concept of HCM^16^, using pedestrian space as the dependent variable which was influenced by factors such as the volume-to capacity ratio of pedestrians, volume-to-capacity ratio of vehicles, pedestrian crossing time and parking factor. A videographic technique was used to collect data on pedestrian speed, crossing time, pedestrian, and traffic volume. This was an improved technique over HCM to effectively evaluate the PLOS in a mixed traffic condition in the Indian context. Cepolina et al.^10^ used the concept of the Voronoi local density method and the loss of personal space to evaluate the PLOS as the comfort experienced by pedestrians in pedestrian infrastructures. The Voronoi area is considered as the personal space around the individual and the space available at disposal is called the available space and the required space for a pedestrian decides if the pedestrian experienced comfort at any point. The PLOS was calculated as the aggregate of the loss of required space of all individuals in that area in consideration. The local density method helped to find the dis homogeneity in the comfort experienced by the pedestrians which cannot be explained by other models. Jia et al.^20^ experimented on the effect of local densities and velocity on the comfort and congestion experienced by the pedestrians in a room egress scenario. The data on the pedestrian trajectory was collected through a video analysis and the perceived comfort of pedestrians was collected through a questionnaire survey. It was found that the personal velocity of pedestrians affected the pedestrian’s comfort more than the four local densities such as counting density, Voronoi density, Voronoi-circle density, and Voronoi-semicircle density. The position of pedestrians in the crowd, the position of obstacles, age, and gender affected the congestion experienced by the pedestrians, and the findings could be used to design effective pedestrian infrastructures.

### Quality-based models

Further to quantitative variables such as speed, volume, and flow rate of pedestrians the qualitative variables relating to walkway features and flow characteristics focusing on the quality of service provided by walkways were analysed by collecting data on pedestrians’ perception through questionnaire surveys and field measurements. Jaskiewicz^21^ proposed a trip quality method based on nine measures analysing the safety, aesthetics, and ease of movement which are as follows: enclosure, the complexity of path network and spaces, building articulation, transparency, buffers, shade, trees, overhangs, and physical condition. The components were rated on a scale of 1 to 5 where 1 stands for very poor and 5 stands for excellent and the final PLOS value is the average of these scores on the scale of A to F. Another similar model was proposed by Gallin^22^ based on physical, location and user factors that comprised eleven variables altogether under the three categories. Each of these factors was given a score and the final values of PLOS were based on the aggregated score, showing which variables contributed to the higher or lower LOS and highlighting the condition of Australian roads. Asadi-Shekari et al.^23^ used a point-based system to measure the PLOS by comparing the existing condition of street facilities to the standard guidelines. They considered factors such as traffic speed, buffers and barriers, traffic lanes, midblock crossing, landscape and trees, fire hydrant, furniture, footpath pavement, markings, corner island, sidewalk on both sides, advance stop bar, width of footpath, driveway, lighting, signage, bench, drinking water, signal, sidewalk grade, ramps, warning tile, tactile pavement, curb ramp, wheelchair accessible drinking fountain and slope. There could be more variables that may have to be included and some may not be relevant and must be removed while measuring the PLOS of the walkways using this method at a different location. Talavera-Garcia and Soria-Lara^24^, created an index to measure the quality of PLOS (Q-PLOS) by choosing factors for accessibility, safety, comfort, and attractiveness by conducting a pedestrian preferences survey. The weights were assigned to these variables and their quality values along with weights were applied to the formula for Q-PLOS. The accessibility to the nearest amenities such as public transport stops could be analysed using this method. Bivina and Parida^44^ considered factors related to safety, security, mobility and infrastructure, and comfort and convenience to find the PLOS of sidewalks in Indian cities using structural equation modelling (SEM). The pedestrian ratings of the different features were collated through surveys and exploratory factor analysis was used to retain the significant factors and to relate them to the PLOS as latent exogenous and endogenous variables. Vallejo-Borda et al.^25^, developed a similar SEM model by collecting pedestrian perception on thirty-two factors relating to sidewalk characteristics, externalities, surroundings, discomfort, bike hassles, protection, and amenities. They did exploratory factor analysis to find the effect of the latent variables on the Quality of service (QoS) of the sidewalks and found the sidewalk characteristics directly affected the QoS, while the other latent variables indirectly affected the pedestrian perception of the QoS.

### Performance-based models

In the evaluation of the walkways using PLOS, pedestrian perception of safety and comfort were the focus of some of the methods which are grouped under the performance-based models. Landis^26^ developed a stepwise multivariable regression model by choosing the variables that significantly affect the safety and comfort feeling of pedestrians. A survey was conducted, and it was found that the pedestrians considered speed and travel time, freedom to manoeuvre, traffic interruption, and convenience of the facility as the most influencing variable which was used in the mathematical expression to find the PLOS. Zhao et al.^27^ proposed a fuzzy neural network model by considering multi-type factors based on traffic conditions, road facility conditions, and environmental conditions and correlating them to pedestrian safety and comfort satisfaction scores on a scale of 1–10. The influencing factors for the model were selected using the Spearman rank correlation method and this method considered the visual satisfaction of the pedestrians using that footpath. Shu et al.^28^ followed a similar method of evaluating pedestrian safety and comfort using a fuzzy neural network to develop a PLOS model for different pedestrian flow rates. At traffic intersections, the safety score, convenience score, and efficiency score of pedestrians were combined to find the PLOS using a fuzzy linear regression model. The factors considered were pedestrian, traffic, and intersection characteristics which were collected using pedestrian surveys and video recordings, and the significant variables were selected using Pearson’s correlation^29^. The crossing LOS was evaluated by collecting data on pedestrians’ perceived LOS while crossing the road and using the ordered probit model by Kadali and Vedagiri^30^. The significant variables used in the model were land use type, crossing difficulty, crossing safety, width of median, number of lanes, and number of vehicles which were selected from low p-values. The use of land use types considered in this study helped to evaluate the tendency of people to use crosswalks at different locations. The PLOS of sidewalks under mixed traffic conditions was modelled by obtaining data about the overall satisfaction of pedestrians while walking on them, using multiple variable regression^7^. Width of walkways, pedestrian volume, traffic volume, and lateral separation were the variables included in the model. Recently, perceived LOS was modelled by conducting questionnaire surveys, and the ordinal regression model was used to relate the perceived LOS to age, gender, perceived comfort, and street characteristics^6^.

There has not been much work done on the pedestrian level of service in employing both quantitative and qualitative variables that influence the PLOS. It has been found from previous research that pedestrian comfort varies according to how far a pedestrian walks how often they use a particular walkway and what the purpose of the trip is. Hence in this study, the PLOS model has been developed for three main trip purposes such as education, work, and recreation by using pedestrian flowrate and all possible qualitative variables relating to path characteristics, pedestrian characteristics, and flow characteristics that impact the pedestrian feeling of comfort in using a particular footpath. The machine learning model, constructed using Light GBM and Random Forest, has been elaborated upon using a game theory-based method known as SHAP. This technique elucidates the impact of each factor on the Pedestrian Level of Service (PLOS) during a specific prediction instance. Moreover, SHAP facilitates a comprehensive interpretation of tree-based models on a global interpretation.

## Dataset

**Figure 1:**
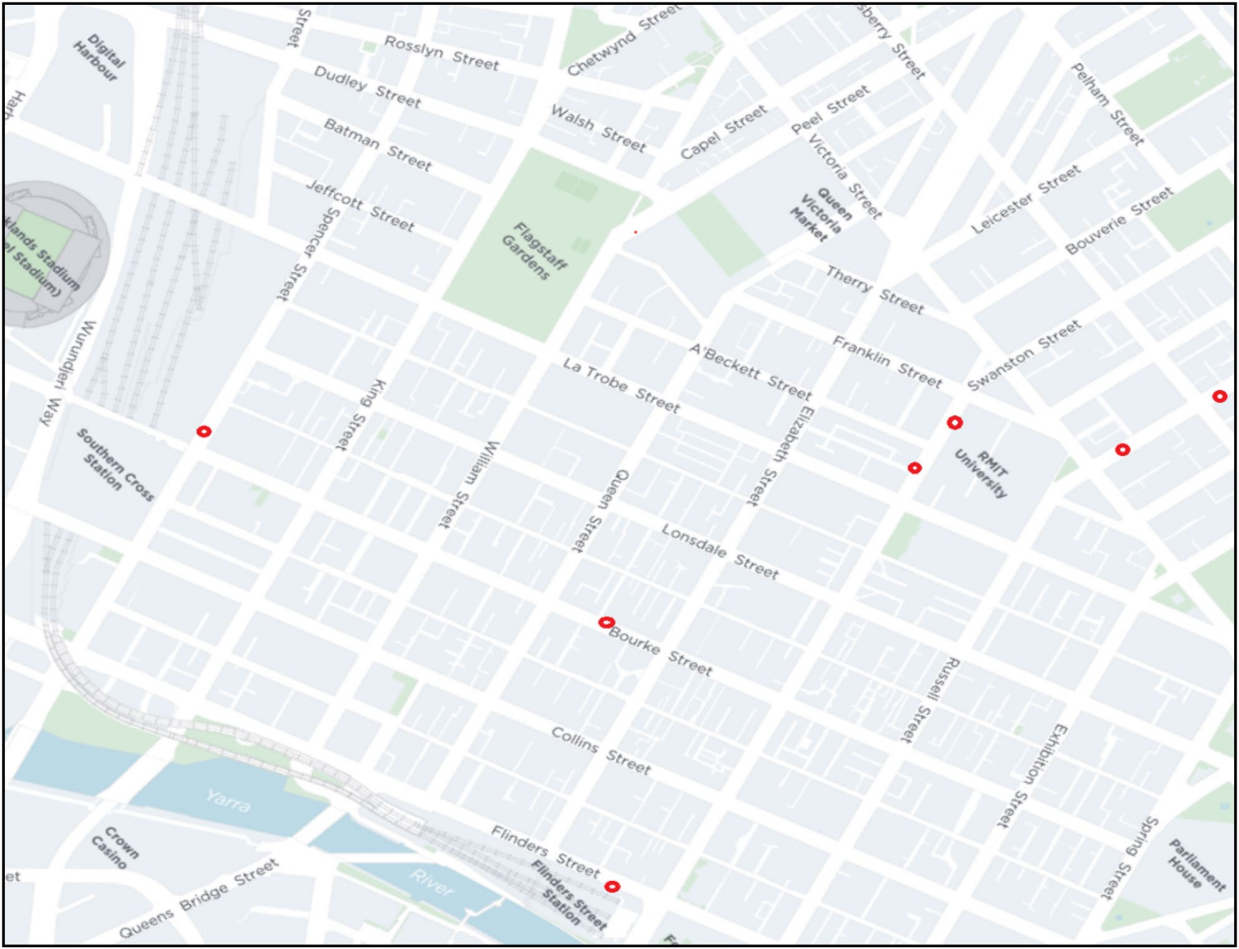
Map showing the survey locations in Melbourne’s central city.

Data for this study was gathered within Melbourne's Central Business District (CBD) through questionnaire surveys and pedestrian sensors strategically positioned throughout the city. Before initiating the surveys, ethical approval was obtained from the Human Research Ethics Committee of RMIT University. Pedestrians were informed of the survey’s purpose, and their informed consent was secured before conducting the surveys. In the CBD, a total of 61 sensors were deployed at various locations. For this specific research, seven sensor locations were chosen: RMIT Building 14, RMIT Building 80, Southern Cross Station, Flinders Street Station, Bourke Street Mall, Lygon Street East, and Lygon Street West (refer to Fig. 1). RMIT buildings were frequented by students on educational trips, Southern Cross and Flinders Street stations were primarily used by daily commuters, and Bourke Street, Lygon Street East, and West were selected due to the recreational activities in those areas. This selection aimed to capture responses from pedestrians engaged in these specific trip purposes. The sensors provided data on the hourly pedestrian flow rate within the one-meter width of the walkways.

### Survey data

The qualitative data used for the research was collected through an intercept survey where the pedestrians walking on the footpath were randomly selected, explained the purpose of the survey, and asked to provide their opinion on comfort if they were interested. The survey was conducted only according to the guidelines mentioned by the Human Research Ethics Committee about human research. The surveys were conducted from 7:30 am to 4:30 pm to accommodate peak and off-peak flow of pedestrians. The total valid responses collected, and which were used for the model was 663 which included all three trip purposes. The minimum sample size required was calculated using the formula referred by Naing et al.^31^, as follows:

1 $$n=\frac{N {\times} \upchi 2 {\times} p\times(1-p)}{{\varepsilon }^{2} {\times} \left(N-1\right)+\upchi 2 {\times} p {\times} (1-p)},$$

where n- is the size of the sample to be calculated; N – the size of the population; $\upchi 2$ – Value of chi-square at the expected confidence interval; p – the expected proportion of the population; ε – margin of error.

The following values were used for estimating the sample size:

N = 159,810, $\upchi 2$ = 3.84 (at 5% confidence interval), p = 0.2, $\upvarepsilon$ = 0.05, which gives the value of n = 245.

The total responses collected were well above the minimum samples required for the population in the CBD. Out of the 663 completed surveys, 206 were for education, 253 were for work and 204 were for recreation trip purposes.

### Questionnaire

The survey questionnaire was designed to have questions on pedestrians’ feelings about the pedestrian infrastructure, environment, and pedestrian flow characteristics^8,32–34^. The questionnaire could be divided into five sections as follows:

### Socio-demographic factors

The questions under this comprise, the pedestrian:

i. Age group – 0–18 to 35; 1–36 to 50; 2–51 to 65; 3- > 65.
ii. Gender – 0-Male; 1- Female.
iii. Trip purpose- 1-Education; 2-Work; 3-Recreation; 4- Other.
iv. Comfort distance from others – 0-0 m to 1 m; 1- 1 m to 1.5 m; 2–1.5 m to 2 m; 3—> 2 m.
v. Follow social distancing – yes or no.

### Dynamic factors affecting comfort

These questions are the pedestrian’s feeling of comfort about:

i. Pedestrian crowd at that moment -1-Very uncomfortable; 2-Uncomfortable; 3-Neutral; 4-Comfortable; 5-Very comfortable.
ii. Vehicle volume on the road close to them at that moment -1-Very uncomfortable; 2-Uncomfortable; 3-Neutral; 4-Comfortable; 5-Very comfortable.

### Pedestrian and flow characteristics

These questions include the pedestrian’s opinion on:

i. Slow-moving pedestrians do not obstruct the footpath- 1-Strongly disagree; 2-Disagree; 3-Neutral; 4-Agree; 5-Strongly agree.
ii. Opposite direction flow of pedestrians is minimum—1-Strongly disagree; 2-Disagree; 3-Neutral; 4-Agree; 5-Strongly agree.
iii. Can maintain personal space while walking—1-Strongly disagree; 2-Disagree; 3-Neutral; 4-Agree; 5-Strongly agree.
iv. No high speed and noisy traffic on the road next to them—1-Strongly disagree; 2-Disagree; 3-Neutral; 4-Agree; 5-Strongly agree.
v. Maintain COVID-19 safe social distance -1-Strongly disagree; 2-Disagree; 3-Neutral; 4-Agree; 5-Strongly agree.

### Path characteristics

These questions included pedestrians’ opinions about the width of the footpath, continuity of the footpath, street furniture, surface of the footpath, lighting, buffers, non-slippery footpath, landscaping, street benches, street vendors, on-street parking, detours, and construction sites blocking the footpath. They were rated on a Likert scale of 1 to 5 where 1 denotes strongly disagree and 5 denotes strongly agree.

The final question is about the pedestrian’s perception of the overall comfort of walking on the footpath which is taken as the PLOS was given ratings on a Likert scale of 1–5. Where 5 stands for A which is excellent and 1 stands for E which is very poor LOS.

In summary we see that the variables used for the study are Age group, Gender, Comfort distance, Pedestrian crowd, Vehicle volume, Continuous footpath, Footpath width, Street furniture, Footpath surface, Lighting, Buffers, Non-slippery, Landscaping, Street benches, On-street parking, Slow-moving pedestrians, Opposite direction flow, Personal space, Highspeed traffic, Detours, Construction sites, Covid safe distance and Overall comfort of walking (PLOS) on the footpath which are all qualitative variables. The pedestrian flow rate data taken from the sensor is taken as the quantitative variable. Overall, for three trip purposes, the proportion of data collected for the pedestrian level of service 1 to 5 was 3% for Very Poor (LOS E), 12% for Poor (LOS D), 32% for Average (LOS C), 42% for Good (LOS B) and 11% for Excellent (LOS A) condition of Level of service. Since there were very few people rating the LOS to be very poor, the LOS E (Very poor) has been combined with LOS D (Poor) in the model, to avoid underfitting^6,30^.This is consistent with the previous research where the PLOS has been reduced to four categories because pedestrians find it difficult to classify a midblock footpath on a scale of 1 – 5. A description of the PLOS categories used in the research is shown in Table 1.

**Table 1:** Description of the target variable used in the model.

PLOS	Rating	Description	Education	Recreation	Work
A	5	Excellent	6%	15%	12%
B	4	Good	45%	44%	36%
C	3	Average	34%	26%	34%
D	1&2	Poor	15%	15%	18%

**Figure 2:**
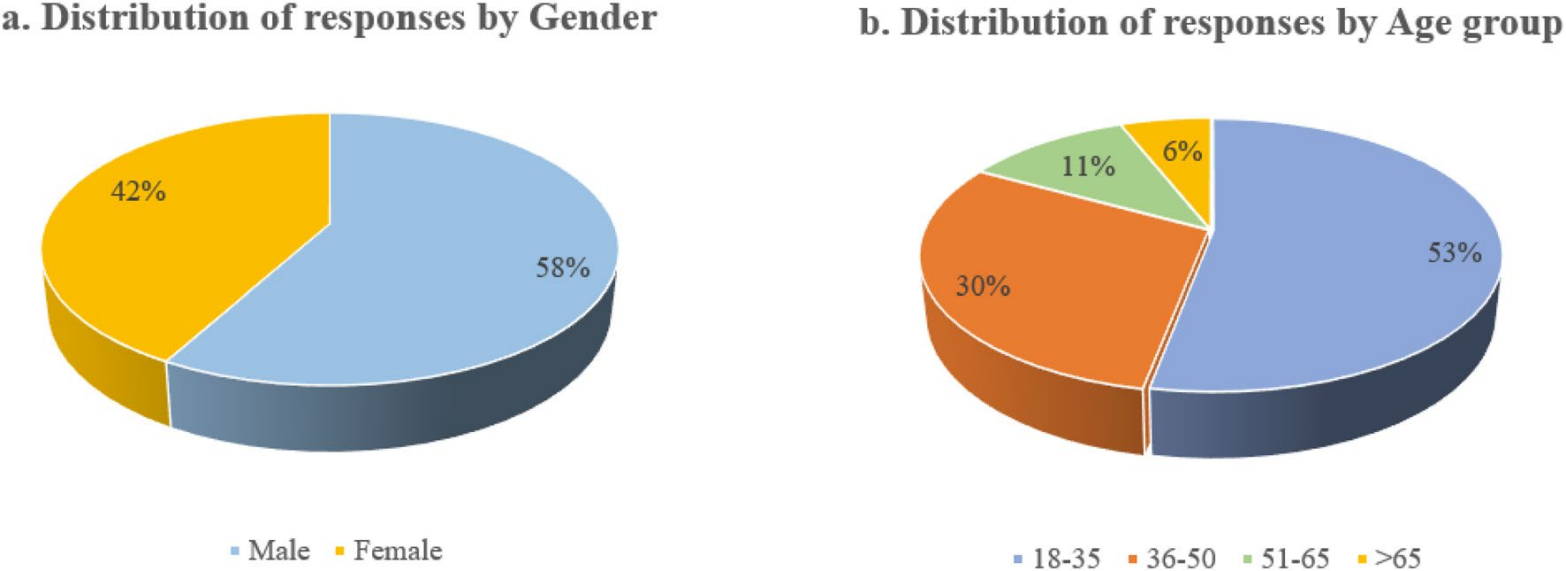
(**a**) Distribution of total responses by gender (**b**) distribution of total responses by age group.

The survey was carried out in the city centre by approaching pedestrians randomly on selected walkways as shown in Table 2. Only those who provided their immediate consent were included in the study. Individuals using wheelchairs or pushing prams were excluded due to potential mobility restrictions, which might introduce additional variables for consideration in the study. The demographic distribution of participants is illustrated in Fig. 2a and b, revealing that 58% of respondents were men, while 42% were women, aligning with the proportional representation of males and females in central Melbourne. Although an 'others' category was available in the survey, very few respondents identified themselves as such for gender, and their data was treated as outliers and removed. Approximately half (50%) of the respondents fell within the 18–35 age group, primarily consisting of individuals on 'education' and 'work' trips. The 36–50 age group contributed to 30% of the completed surveys, with the remaining 17% comprising participants above 50 years of age. Regarding the comfort distance from other pedestrians, direct responses indicated that about 84% of individuals preferred a distance less than 1.5 m around them, while only 16% favoured a comfort distance greater than 1.5 m.

**Table 2:** Responses collected from survey locations.

S. No	Locations	Number of responses	Average pedestrian flowrate (ped/m/hr)	Ratio of responses to flowrate
1	Flinders street station	170	1147	0.148
2	Southern cross station	132	661	0.199
3	RMIT 14	29	820	0.035
4	RMIT 80	183	1480	0.123
5	Bourke street	40	1511	0.026
6	Lygon street east	96	980	0.098
7	Lygon street west	34	462	0.073

## Methodology

### Mutual information gain

It helps to find out the interdependence of two random variables. It is the measure of the amount of information one can get about a random variable given another random variable. The value of mutual information gain between two variables is zero if the variables are independent. The higher the value of mutual information, there is more dependence between both variables.

Mutual Information (MI) between two variables A and B can be stated as:

2 $${\text{MI }}\left( {{\text{A}};{\text{ B}}} \right) \, = {\text{ H}}\left( {\text{A}} \right) \, {-}{\text{ H}}\left( {{\text{A}}|{\text{B}}} \right),$$

where H(A) is the entropy of A.

And H(A|B) is the conditional entropy of variable A when variable B is given. Because it measures the dependence of two variables,

3 $${\text{MI }}\left( {{\text{A}};{\text{ B}}} \right) \, = {\text{ MI }}\left( {{\text{B}};{\text{ A}}} \right).$$

In this study, the mutual information gain is used to find the mutual dependence of the variables affecting pedestrian comfort and the PLOS. The variables that give a value of zero have not been included in the model.

### Random forest algorithm

A Random Forest is a tree-based machine-learning algorithm that is used for regression and classification problems. A random forest algorithm is a forest of decision trees using bootstrap aggregating. This is an extension of the usual bagging method for regression or classification. The bootstrap samples are data samples created from a training data set using sampling with replacement. In ensemble learning methods, several base models are combined using simple methods such as averaging or majority voting or by using complex methods such as boosting or stacking.

**Figure 3:**
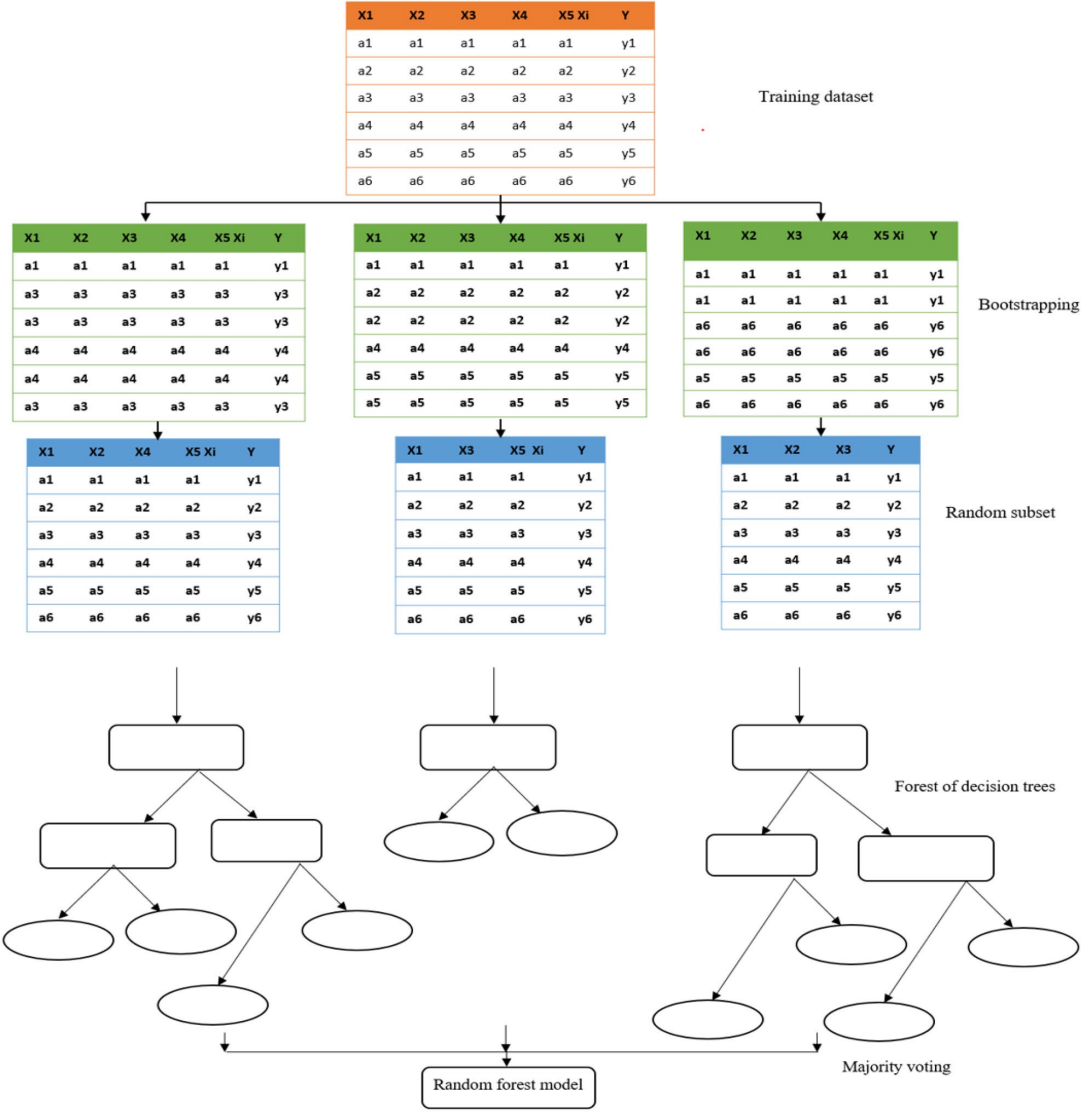
Illustration of random forest model^35^.

Random forest algorithm minimizes overfitting by creating models that are not correlated by giving different training data sets and by averaging the models. To further reduce the correlation between the trees it uses different randomly selected features from the training data as shown in Fig. 3. This can be achieved by changing the hyperparameter of individual trees, which is max_features.

Thus, in the random forest algorithm, each decision tree is trained on different data sets that have different numbers of rows and columns/features. Finally, the decision trees are combined by majority voting i.e., the most frequent categorical variable for classification problems and averaging outputs for regression problems. One-third of the training sample drawn from the training set with replacement is set aside as test data also known as out-of-bag sample (OOB). The prediction error of training samples is found using these test data. The hyperparameters of the random forest model can be tuned to prevent overfitting and increase the predictive power of the model. Some of the hyperparameters are maximum depth, minimum samples per split, maximum terminal nodes, minimum samples per leaf, number of estimators, maximum number of features, and maximum samples.

### Light GBM

Light GBM is also a supervised machine learning technique that is decision tree-based with a gradient-enhancing framework. It uses two new techniques: Gradient-based side sampling (GOSS) and exclusive feature bundling (EFB)^36^. These techniques improve the efficiency, scalability, and memory usage of the model. In GOSS, the data instances are sorted according to the absolute value of gradients, and the top p × 100% instances are chosen and then from the remaining data q × 100% instances are sampled randomly. When calculating the information Gain, GOSS amplifies the sampled data with small gradients, by a constant $\frac{1-{\text{p}}}{{\text{q}}}$^36^. In EFB, the mutually exclusive features that take nonzero values in sparse feature space are bundled, where the number of bundles will be less than the number of features and it improves the training speed of the Light GBM model.

**Figure 4:**
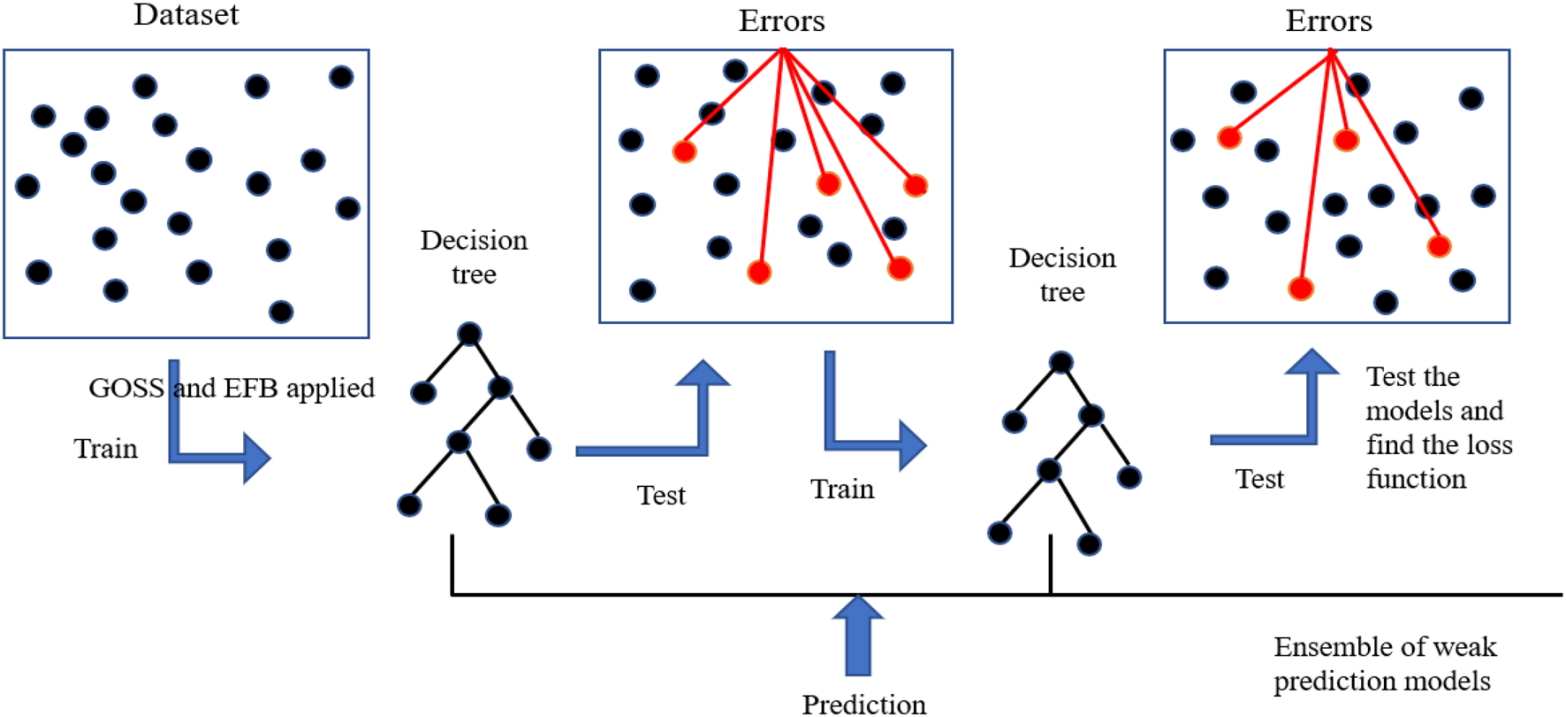
Illustration of Light GBM model creation^37^.

Light GBM trees grow leaf-wise contrary to other gradient-boosting algorithms that splits the tree level-wise. It enables the leaf with the maximum delta loss to grow. Since the leaf is fixed based on maximum depth, the leaf-wise growth is small compared to the level-wise growth of other algorithms. The working of the Light GBM algorithm with the application of GOSS, EFB, and leafwise split is illustrated in Fig. 4.

### SHAP

SHAP stands for SHapely Additive exPlanations, a framework based on game theory, which explains the outcome of a machine learning model. It has been developed by Scott-Lundberg and Su-In^38^. With the increase in machine learning algorithms and the use of disparate data sources and large input features, the results produced are “black box” solutions that cannot interpret and explain themselves. Interpretability stands for how a machine learning model can relate an input feature to an output and Explainability stands for how a machine learning mechanism be explained in human terms that could be understood. SHAP helps to find the feature importance of the input variables. SHAP values are generated for each input feature, which is calculated as the change in expected model prediction concerning that feature. The SHAP value given by the following equation is the value calculated for each feature,

4 $${\text{y}}_{{\text{j}}} = {\text{ y}}_{{{\text{base}}}} + {\text{f}}\left( {{\text{X}}_{{{\text{j1}}}} } \right) \, + {\text{ f}}\left( {{\text{X}}_{{{\text{j2}}}} } \right) \, + {\text{ f}}\left( {{\text{X}}_{{{\text{j3}}}} } \right) \cdots + {\text{f}}\left( {{\text{X}}_{{{\text{jk}}}} } \right).$$

The above equation represents a row in a two-dimensional SHAP array. j stands for the rows and k stands for the final column of the two-dimensional array. The row represents the predictor features (X_1,_ X_2,_ X_3_, etc.) of the response variable and the ‘y’ column has the predicted PLOS value corresponding to that row of SHAP values. The value of f(X_j1_) is the SHAP value of the variable Xj. Each ‘f’ function relates to the variation in the average predicted PLOS value from the actual observed PLOS. If f(X_jk_) is greater than zero then it increases the predicted probability and if less than zero, then it reduces the predicted probability of PLOS.

To understand the results of the Random Forest model in this study, the following SHAP value plots will be used:

1. Variable Importance plot – this plot helps to list the features in decreasing order of importance. The features at the top are the most important and those at the bottom are the least important variables.
2. Summary Plot – this plot is like a variable importance plot as the features are listed in their order of importance from top to bottom. But it represents these features for each class or category of response variable.
3. Waterfall plot – this shows the influence of features on the predictor variable at a particular or single instance.

### Model assessment

The random forest and Light GBM model use 80% of the data for training the model. The accuracy of prediction using these models can be evaluated using the metrics calculated from the confusion matrix. The remaining twenty percent of data is used to test the performance of the models. The values taken from the confusion matrix are used to find the following four measures 1. Accuracy 2. Precision 3. Recall and 4. F-score. These measures can be represented as follows:

5 $$Accuracy = \frac{True \,\,Positive}{True\,\, Positive+False \,\,Positive+True \,\,Negative+False \,\,Negative},$$

6 $$Precision = \frac{True \,\,Positive}{True \,\,Positive+False \,\,Positive},$$

7 $$Recall = \frac{True \,\,Positive}{True \,\,Positive+False\,\, Negative},$$

8 $$F1\,\, score\boldsymbol{ }= 2 \times \frac{Precision {\times} Recall}{Precision+Recall}.$$

Accuracy^39,40^ (Eq. 5) is the most common and simple metric used to find if the model is working well for the given data. But relying only on accuracy can be misleading specifically for an imbalanced class of data. The complement metric of accuracy is misclassification error rate which is given as the ratio of negative predictions to the total number of predictions. Precision (Eq. 6) gives the measure of the performance of positive instances. Recall (Eq. 7) also known as Sensitivity or True positive rate gives the value of true positive to the total number of actual positives. Finally, the F1 score (Eq. 8) gives the balanced metric of both precision and recall for the imbalanced class of data as it is based on both measures.

## Results

The models are developed using Random Forest and Light GBM algorithms which also include testing for prediction accuracy. The choice of the 80–20 percent split in the use of data corresponds to the training set and the test set respectively as explained before. The metrics such as accuracy, precision, recall, and f-score are used to evaluate the performance of models which is shown in Table 3. Due to a smaller number of pedestrians rating the LOS of roads as 1 or very poor, the data corresponding to ratings 1 and 2 has been combined as ‘1’. Hence the five levels of service are reduced to four and the percentage of data collected for each class and trip purpose is shown in Table 1. While explaining the feature analysis and working of the model, class 3 represents PLOS A, class 2 represents PLOS B, class 1 stands for PLOS C, and finally class 0 stands for PLOS D.

**Table 3:** Metrics of the machine learning models.

Classifier/model	Random forest			Light GBM		
Trip purpose	Education	Recreation	Work	Education	Recreation	Work
Train						
Accuracy	0.65	0.69	0.65	0.67	0.77	0.62
Precision	0.66	0.74	0.74	0.68	0.77	0.64
Recall	0.65	0.69	0.65	0.67	0.77	0.62
F1-score	0.62	0.66	0.63	0.66	0.77	0.62
Test						
Accuracy	0.64	0.76	0.71	0.74	0.80	0.70
Precision	0.66	0.74	0.72	0.71	0.81	0.72
Recall	0.64	0.76	0.71	0.74	0.80	0.70
F1-score	0.60	0.74	0.71	0.71	0.81	0.68

From Table 3 it is observed that the Light GBM model performs more accurately than the Random Forest model for Education and Recreation trip purposes. It works out to be 10% and 5% more accurate than the Random Forest model by giving accuracy scores of 0.74 and 0.80 and f1-scores of 0.71 and 0.81. The sensitivity of the models seems to be slightly higher for Education and Recreation trip purposes when evaluated using Light GBM models. The accuracy, sensitivity, and f1- scores of the models for Work trip purposes are almost the same for both the machine learning models. To further estimate the predictive capability of the two machine learning models the AUROC (Area under the receiver operating characteristic curve) value can be used^41^. The area under the curve gives an idea of how well a model can help to identify the classes of a response variable. In multiclass classification, the AUC curve is determined by binarizing the output as one vs rest or one vs one. In this case, one vs rest is used to find the value of AUROC which is shown in Table 4. It shows that the AUROC values are slightly higher for the Light GBM models for all three trip purposes. Hence the light GBM model will be analysed further using a model agnostic method based on game theory, called SHAP to understand its features and the results.

**Table 4:** ROC-AUC values for the different models.

Classifier/model	Trip purpose	AUROC (one-to-rest)
Random forest	Education	0.729
	Recreation	0.873
	Work	0.854
Light GBM	Education	0.838
	Recreation	0.891
	Work	0.862

### Feature importance using SHAP

The SHAP summary plot serves as a tool to elucidate the features that hold greater significance in predicting the pedestrian level of service (PLOS) within the model. Additionally, it aids in identifying features that specifically influence a particular class, offering a comprehensive, global interpretation of the model. On this plot, the Y-axis denotes the feature names arranged in descending order of importance, reflecting their impact on the model's output. Meanwhile, the X-axis represents the mean absolute Shapley values, providing a measure of the magnitude of feature attributions. This graphical representation offers a clear visualization of the relative importance of features in shaping the predictions and understanding the model's overall behaviour.

**Figure 5:**
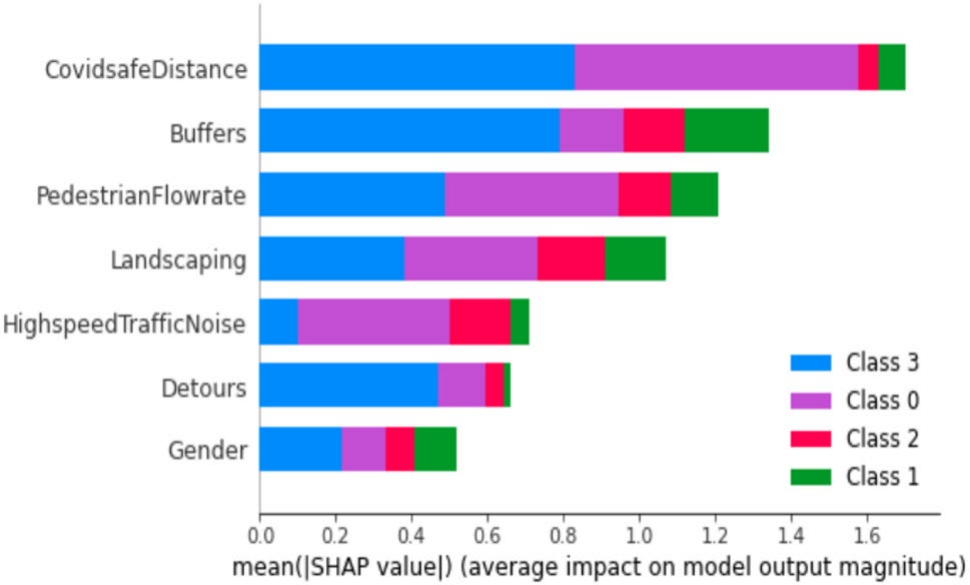
Feature importance using Shapley values for education trip purpose model.

**Figure 6:**
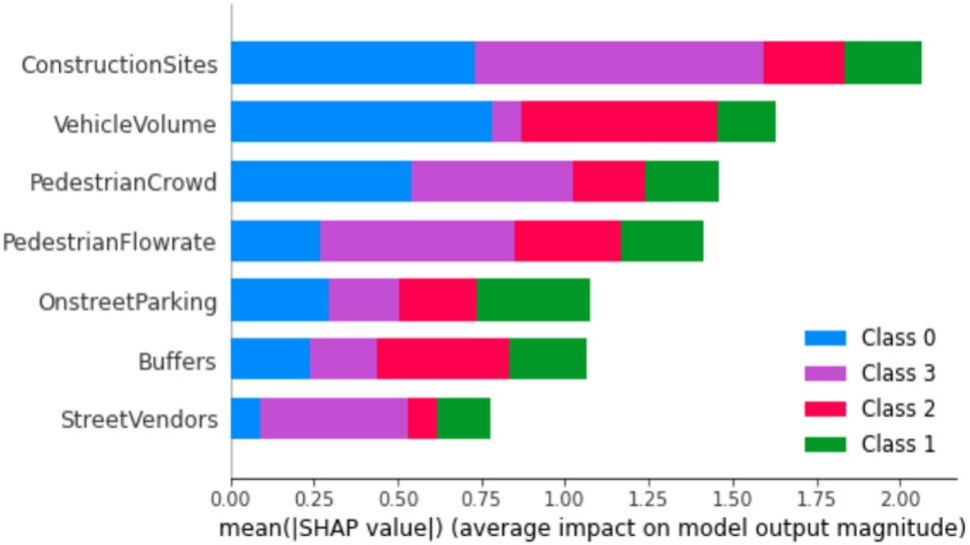
Feature importance using Shapley values for recreation trip purpose model.

**Figure 7:**
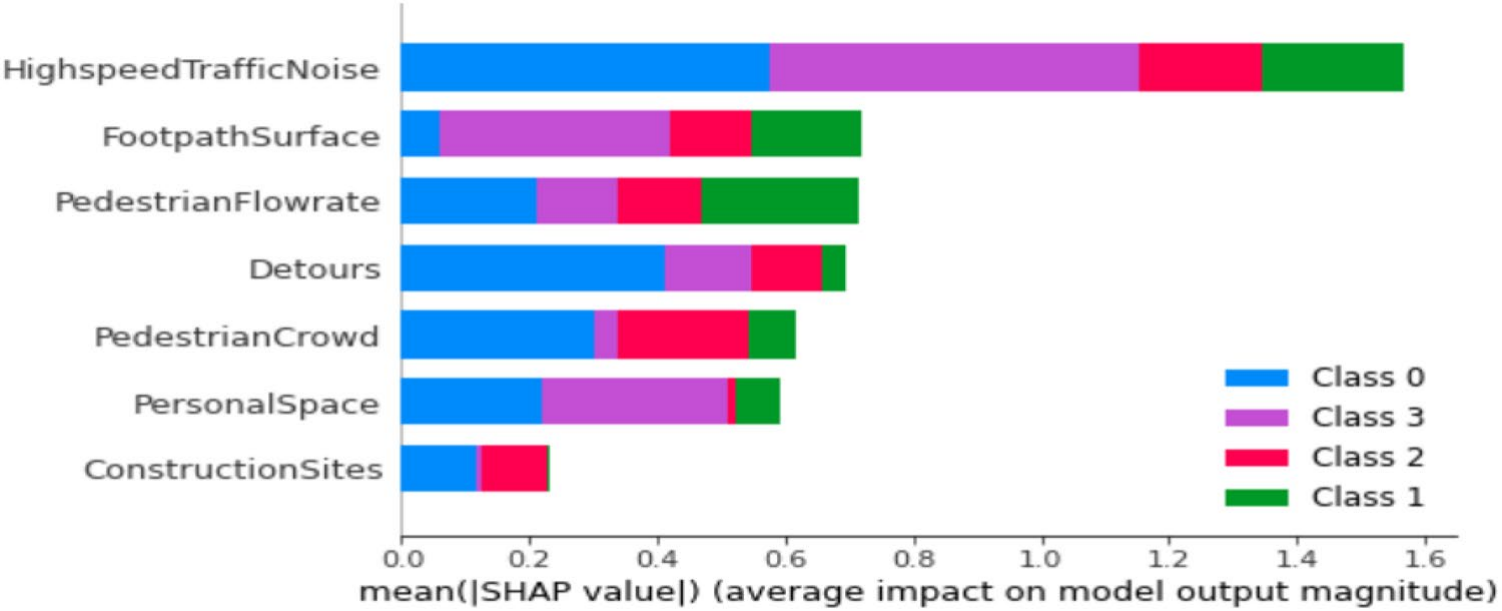
Feature importance using Shapley values for work trip purpose model.

Lundberg and Lee^42,43^ discovered that commonly employed methods such as feature importance permutation and a mean decrease in impurity fail to meet the criteria of consistency and accuracy. These criteria entail that the cumulative contributions of each feature should equate to the total contribution in the model. The feature importance of models for three trip purposes is depicted in Figs. 5, 6, and 7. Notably, the pivotal features for educational trips, as indicated in Fig. 5, encompass interpersonal space between pedestrians, safe distance from vehicles on the road, and pedestrian speed. These factors significantly contribute to predicting the four categories of PLOS. Conversely, for recreational trips, Fig. 6 reveals that PLOS is chiefly influenced by construction sites, vehicle volume, and pedestrian crowd. Numerous locations in the central city witness detours caused by construction zones, particularly during pedestrian walks.

Figure 7 illustrates that individuals walking in the city for work-related purposes prioritize certain factors in the prediction of PLOS. These factors include traffic noise, footpath surface, detours, and pedestrian speed. Notably, construction sites only impact PLOS A and B without affecting other levels of service. This model provides valuable insights into the behaviour of pedestrians traversing city walkways, effectively capturing preferences based on different destinations.

The model effectively reveals distinct preferences among pedestrians. For instance, students walking to universities focus primarily on maintaining a suitable distance from others and vehicles, along with the ability to walk at a pace conducive to reaching their classes on time. Pedestrian density and vehicle volume are of lesser concern to them. It's worth noting that educational sites, where the data was collected, experience a significant surge in pedestrian volume during daytime class hours. On the other hand, pedestrians strolling for recreational purposes express discomfort with high pedestrian density, substantial vehicle volume, and interruptions caused by construction activities on footpaths. These factors hinder the enjoyment of their walk on the footpaths. For commuters heading to work, footpath usage is routine, and they are acutely aware of pedestrian density and vehicle volume during their typical travel times. Their concerns include traffic noise on busy roads, a desire for a clean footpath surface free of spilled food or trip hazards, and apprehension about unexpected detours that might extend travel time and alter walking speed on footpaths.

### SHAP summary plot

The influence of a feature on the class is aggregated to create a plot illustrating the feature's importance for that class. In this summary plot, each point corresponds to a Shapely value of the feature at a specific instance. The y-axis signifies the ranking of feature importance for the model's output, while the x-axis is determined by the SHAP value, representing the magnitude of change in log odds. In this graphical representation, points are denoted in both blue and red, where blue signifies a low value of the feature, while red indicates a high value of the feature. This colour coding effectively highlights the impact of feature values on the model's output, providing a visual representation of their relative importance.

**Figure 8:**
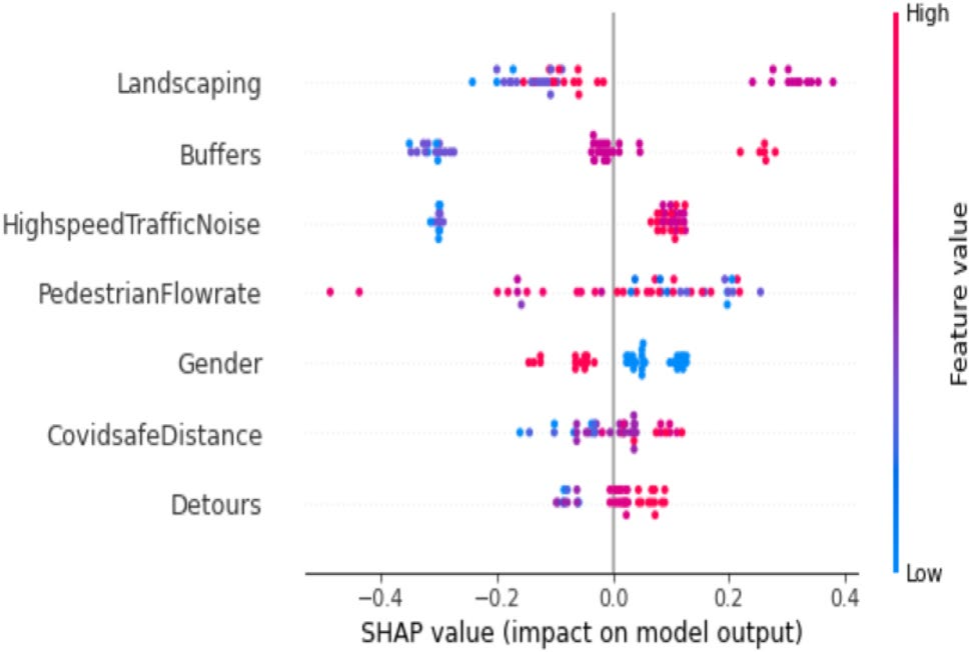
SHAP summary plot for education trip purpose at a good level of service.

Figure 8 highlights the impact of landscaping and minimal disturbance of traffic noise on the pedestrian level of service (PLOS) for footpaths in educational areas. Points marked in red emphasize that higher ratings for landscape and minimal traffic noise directly contribute to an elevated PLOS, indicating a higher level of comfort. Specifically, in instances where a good level of service is observed, pedestrians on the footpath prioritize factors such as maintaining a safe distance from vehicles on the road, minimizing traffic noise, and enjoying a walkway adorned with trees and a refreshing landscape. These considerations underscore the importance of environmental elements in influencing the perceived comfort and quality of footpaths in educational settings.

**Figure 9:**
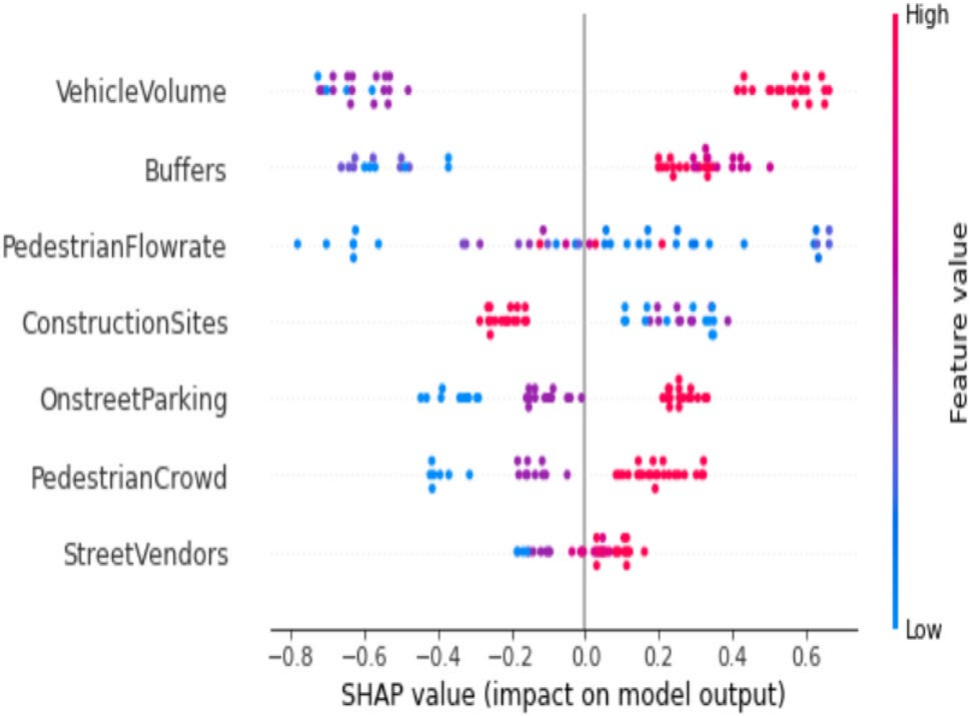
SHAP summary plot for recreation trip purpose at PLOS B on the footpath.

Figure 9 represents the model output for Pedestrian Level of Service (PLOS) in recreational areas, specifically focusing on a good level of comfort corresponding to PLOS B. In instances where a better PLOS is observed, pedestrians express a preference for specific conditions. Pedestrians, aiming for a good PLOS in recreational areas, show a preference for lower vehicle volumes on the adjacent roads. They also desire a safe distance from vehicles traveling on the road. Additionally, a key factor contributing to a favourable PLOS is the absence of disruptions on the footpath caused by construction activities. These insights underscore the importance of factors such as reduced traffic, safety from vehicles, and uninterrupted footpaths in enhancing the perceived comfort of pedestrians in recreational areas.

**Figure 10:**
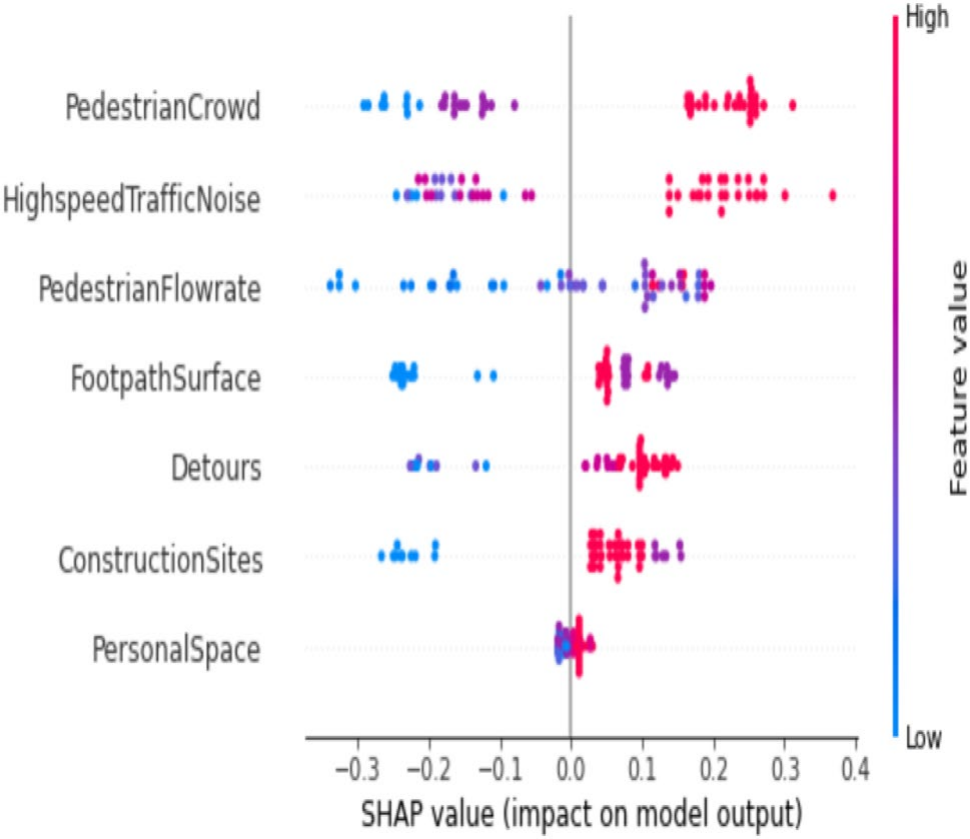
SHAP summary plot for work trip purpose at PLOS B on the footpath.

The inference drawn from Fig. 10, particularly for instances reflecting a good comfort level, indicates that red points, denoting higher comfort ratings, have a positive impact on the Pedestrian Level of Service (PLOS) rating for PLOS B. Specifically, the factors contributing to this positive impact include experiencing lower pedestrian density around them, reduced traffic noise on the road, and the ability to walk at the required speed on footpaths.

The utilization of the SHAP technique across all three trip purposes in deriving this explanation underscores the reliability of the Light GBM model in predicting the Level of Service (LOS). The consistency of these findings across various trip purposes highlights the robustness and generalizability of the model's predictions, reinforcing its trustworthiness in assessing and predicting pedestrian experiences across different scenarios.

### Waterfall plot

So far, SHAP has been used to determine the impact of individual features on model predictions for global interpretation. Additionally, it has been applied to a specific class of LOS for footpaths. SHAP also explains how features affect a single instance of prediction, providing local interpretation. It helps to explain, for a specific class and at a specific row of Shapley values, which variables improve the PLOS and which features reduce the rating of PLOS. SHAP waterfall plots assist in identifying how the model arrived at a prediction for a single observation.

**Figure 11:**
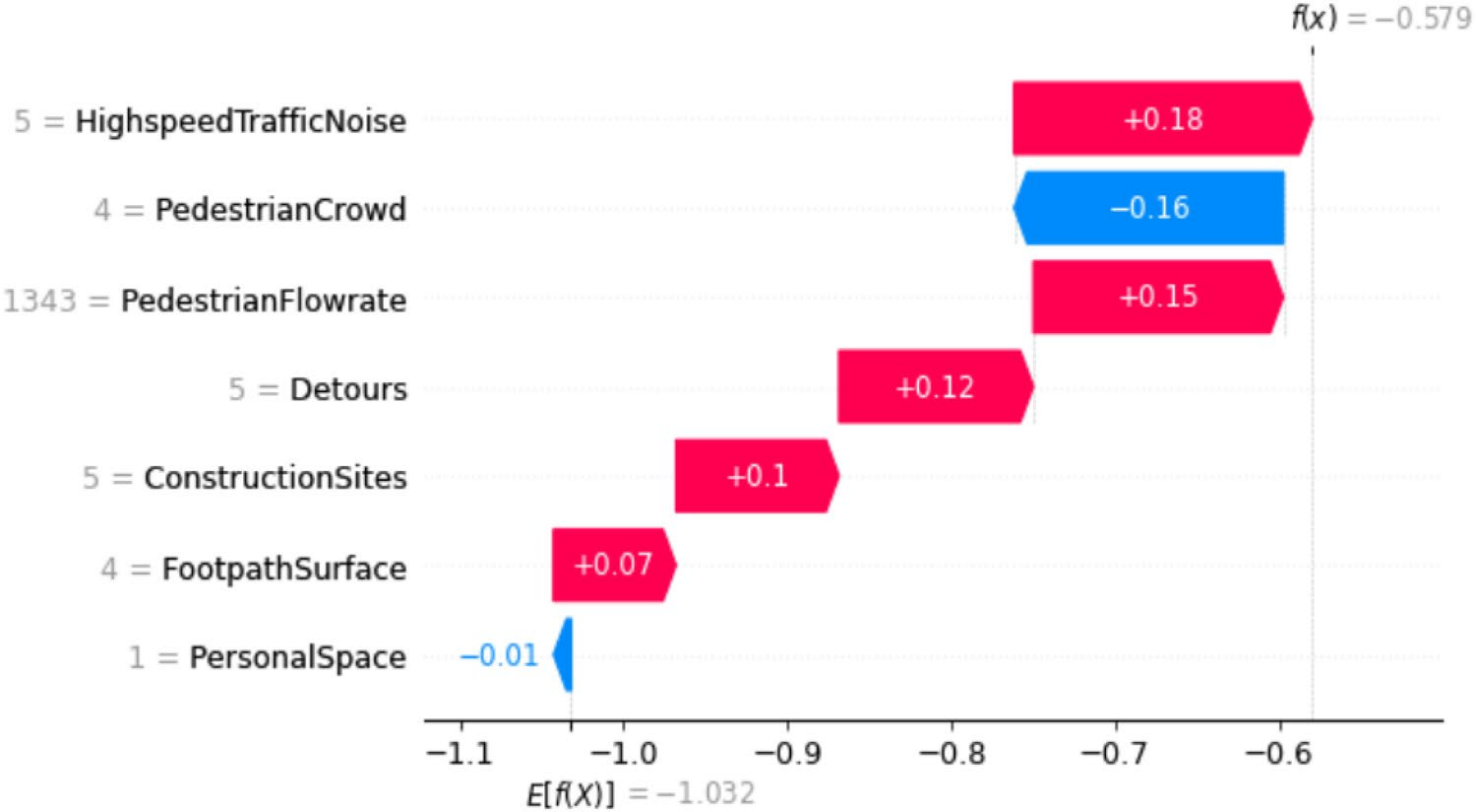
SHAP waterfall plot for a single prediction using the Work trip purpose model.

In Fig. 11, we observe that f(x) represents the model-predicted probability value for LOS (level of service) B, while E[f(x)] is the base value. The values of the features to the left correspond to the actual observations in the data, and the values on the arrows represent the Shapley values of the features. The red arrows indicate a positive effect of the features, increasing the base value, while the blue arrows indicate a negative effect, aiming to reduce the base value to ultimately yield the predicted model value. In this case, we observe that features such as discomfort experienced by the surrounding crowd and the inability to maintain personal space are negatively impacting the PLOS, leading to a reduction in its rating.

## Discussion

The primary determinants influencing PLOS based on trip purposes exhibit noteworthy variations. Specifically, for students engaged in educational journeys, the key factors include interpersonal distance, the presence of buffers separating pedestrian paths from vehicular traffic and walking speed. This is visually depicted in Fig. 5. Students attribute high importance to maintaining their personal space, a factor critical for ensuring both comfort and security within educational environments. Additionally, their concerns extend to safety considerations related to vehicular traffic, emphasizing the significance of buffer space. Furthermore, students express a preference for walking at their own pace or within a continuous flow of pedestrians. These findings align seamlessly with prior research on PLOS concerning sidewalks, where factors such as safety, security, and the rate of pedestrian flow were identified as pivotal elements influencing pedestrian comfort^18,25,44^. The key determinants influencing Pedestrian Level of Service for work-related trips, as highlighted in Fig. 7, encompass the noise generated by high-speed traffic nearby, the condition of the footpath surface, and the rate of pedestrian flow. Individuals commuting to their workplaces demonstrate heightened concern for the cleanliness and slip-resistant quality of footpaths, as well as the feasibility of walking briskly to ensure punctual arrival. The impact of traffic noise is discernible across various age groups, spanning from 18 to 65. Conversely, those undertaking walks for recreational purposes prioritize factors such as the presence of construction sites, pedestrian density, and vehicular volume in their evaluation of the Level of Service, as depicted in Fig. 6. Ongoing metro line construction and other city development activities have resulted in instances where regular footpaths are temporarily inaccessible, necessitating the provision of alternative routes for pedestrians^45^. The pedestrian level of service for individuals walking in the city for recreational purposes is significantly influenced by two dynamic factors: pedestrian crowd and pedestrian volume. These findings align with previous studies conducted without specific consideration of trip purposes. The impact of these factors on individuals engaged in recreational walks underscores the importance of understanding and addressing the challenges posed by varying levels of pedestrian density and overall foot traffic in urban settings^19,27,32,46^.

## Conclusion and future research directions

Evaluation of pedestrian facilities in central cities is crucial for maintaining service quality, as the condition of walking facilities directly impacts individuals' motivation to choose walking as a mode of transport. This research, based on an extensive literature review, focuses on the pedestrian level of service (PLOS) as a metric to gauge the quality of walking on pedestrian footpaths. Recognizing that PLOS varies with the trip purpose of pedestrians, this study considers three main purposes: education, work, and recreation. Various variables are analysed to determine PLOS, encompassing both quantitative factors such as pedestrian flow rate per hour and qualitative aspects like pedestrians' comfort with walkway conditions—width, continuity, surface quality, street furniture, landscape, buffers, lighting, detours, construction sites, pedestrian crowd, vehicle volume, opposite direction flow, slow-moving pedestrians, and personal space. The overall comfort rating, collected through a questionnaire survey involving randomly approached pedestrians in Melbourne city centre, is used as the PLOS metric. Hourly pedestrian flow rates are derived from pedestrian counting sensors during the survey hours.

The study employs the mutual information gain method to identify significant variables affecting PLOS. Notably, it reveals that these variables differ for each of the three trip purposes. To predict PLOS, the research develops a model using machine learning algorithms—random forest and Light GBM. The results indicate that Light GBM proves more efficient in predicting the LOS of pedestrians. For a comprehensive understanding of the model, the study employs SHAP, a game theory-based technique providing both global and local interpretations. The developed model holds practical applications for city planners and transport departments, enabling them to assess pedestrian perception-based comfort levels on footpaths. This information can guide future changes in highly populated cities, enhancing the overall pedestrian experience. To further refine the model, collecting more data and adjusting variables based on local needs could be considered in future research.

## Data Availability

The datasets collected and analysed during the current study are available from the corresponding author upon reasonable request.
